# Profile of the leprosy endemic in the district of Murrupula, Nampula, Mozambique: A population-based study

**DOI:** 10.1590/0037-8682-0321-2022

**Published:** 2023-02-20

**Authors:** Gabriela de Cássia Ribeiro, Daniele dos Santos Lages, Ana Thereza Chaves Lages, Vânia Brito de Souza, Abdoulaye Marega, Francisco Carlos Félix Lana, Manoel Otávio da Costa Rocha

**Affiliations:** 1 Universidade Federal dos Vales do Jequitinhonha e Mucuri, Departamento de Enfermagem, Diamantina, MG, Brasil.; 2 Universidade Federal de Minas Gerais, Escola de Enfermagem, Programa de Pós-Graduação em Enfermagem, Departamento de Enfermagem Materno-Infantil, Belo Horizonte, MG, Brasil.; 3 Universidade Federal de Minas Gerais, Faculdade de Medicina, Departamento de Clínica Médica, Programa de Pós-Graduação em Ciências da Saúde: Infectologia e Medicina Tropical, Belo Horizonte, MG, Brasil.; 4 Instituto Lauro de Souza Lima, Divisão de Pesquisa e Ensino, Bauru, SP, Brasil.; 5 Universidade Lúrio, Faculdade de Ciências da Saúde, Nampula, Moçambique.

**Keywords:** Leprosy, Epidemiology, Public health

## Abstract

**Background:**

Leprosy is a neglected chronic infection caused by *Mycobacterium leprae*, that is curable. The magnitude of the disease and severity of the debilitation it causes renders leprosy a public health problem. This study aimed to analyze the endemic profile of leprosy in the Murrupula district and evaluate the socioeconomic, clinical, and serological profiles of leprosy contacts.

**Methods:**

A cross-sectional study of patients with leprosy diagnosed between 2013 and 2017 and their household and community contacts was conducted in Murrupula District, Nampula Province, Mozambique. Interviews, simplified dermatoneurological examinations, *Mycobacterium leprae* flow (ML Flow) tests, and Mitsuda tests were performed.

**Results:**

Most of the leprosy cases were multibacillary. The patients had some degree of physical disability. ML Flow positivity was more common in household contacts of the patients diagnosed with leprosy and in community individuals who spontaneously presented for testing. In total, 17 patients were diagnosed with leprosy.

**Conclusions:**

This study revealed an active chain of transmission, hidden prevalence, and operational deficiencies in leprosy surveillance and care. The results suggest that the implementation of a public health policy for leprosy prevention and control in Nampula Province is necessary. In future, the possibility of expanding the policy to the entire country should be considered.

## INTRODUCTION

The World Health Organization (WHO) has classified 20 diseases as neglected tropical diseases (NTDs) caused by various agents, such as viruses, bacteria, fungi, parasites, and toxins^1^. NTDs affect more than 1.6 billion people in 149 countries worldwide, mainly in the tropical and subtropical climates of America, Africa, and Asia. These diseases are important indicators of poverty as they mostly affect socially vulnerable populations^2^.

Leprosy, a disease caused by *Mycobacterium leprae*, is considered an NTD. It is characteristically infectious and transmissible. Although curable, leprosy tends to become chronic and has a high potential for disability³. Thus, leprosy is considered a public health problem in the countries where it occurs^1^.

Mozambique is a country in sub-Saharan Africa on the east coast of the continent and ranked fourth in the highest prevalence of leprosy in the world in 2006, with 3,637 newly reported leprosy cases^3^. Due to the high prevalence of the disease, control efforts intensified between 2000 and 2006 in the five endemic provinces of Cabo Delgado, Manica, Nampula, Niassa, and Zambezia, resulting in the establishment of a database for the entire country^4^.

Due to operational difficulties in leprosy control, in 2016, Mozambique was deemed the country with the highest prevalence of the disease in Africa and was among the top five countries with the highest detection rates on the continent^5^. In 2020, the country reported 2,065 leprosy cases, of which 81.4% were classified as multibacillary and 19.3% had grade 2 physical disability. These figures suggest a late diagnosis¹.

The district of Murrupula, which was selected for the study, is part of the province of North Nampula in Mozambique, and has an area of 3,119 km² and a population of approximately 162,000^6^. This region has a high incidence of leprosy, and most are diagnosed late. Between 2010 and 2017, 354 leprosy cases were diagnosed in the district. Of these, 71.5% were multibacillary, 19.7% had grade 2 physical disability at diagnosis, and 9.9% were detected in patients under 15 years old^5^.

A group of Brazilian researchers with expertise in leprosy proposed a project in Mozambique under a Call for Proposal No. 33/2012 (CAPES) "International Program to Support Research and Teaching through International Faculty and Student Mobility-Pro-International Mobility (Capes/AULP)”. One of its objectives was to contribute to the technological and scientific integration of Portuguese-speaking countries in Africa and Asia. Given Mozambique's epidemiological characteristics with respect to leprosy and the prevalence of social problems, it was anticipated that the incorporation of early diagnosis and leprosy control strategies may contribute significantly to structuring the epidemiological surveillance of the disease in the district, and possibly extend the surveillance to other provinces of the country.

The diagnosis of leprosy is essentially clinical^3^. There is no gold standard laboratory test that can detect the bacillus in all clinical manifestations^7^. In 1998, the WHO emphasized the importance of additional technologies for early diagnosis to detect the infection before the appearance of clinical signs and symptoms^8^.

Studies using serologic tests have been developed by several research groups, including the *Mycobacterium leprae* flow (ML Flow) test, a simple and rapid lateral flow test whose results are obtained from serum or whole blood within 5-10 min, without the need for a laboratory or refrigeration, and thus, can be conducted anywhere^9^. 

ML Flow detects specific antibodies against phenolic glycolipid I (PGL-I) present in the walls of *M. leprae*. This result is related to bacillary load and is highly sensitive for detecting multibacillary forms of leprosy^9^. This test is also useful for classifying patients into multibacillary and paucibacillary forms of the disease, which allows for more appropriate treatment. Among the contacts of patients with leprosy, a positive ML Flow test result indicates a higher risk of contracting the disease^10^.

The intradermal Mitsuda test has been used to detect cellular immune responses to *M. leprae*. It is characterized by the formation of an induration at the injection site and reflects the development of delayed hypersensitivity to the bacillus. Although it has no diagnostic value, it aids in clinical classification and prognosis^11^.

This study aimed to analyze the profile of the leprosy endemic in the Murrupula district and evaluate the socioeconomic, clinical, and serological profiles of household and community contacts of patients with leprosy.

## METHODS

This was a cross-sectional epidemiological study. The study sample included patients with leprosy diagnosed between 2013 and 2017, each patient’s contacts inside and outside their household, and community contacts who presented spontaneously and reported possible contact with a leprosy case.

A diagnosis was made in the communities of Carrupeia, Cavina, Morilo, Muaprato, Muchelelene, Murrupa, Nacocolo, Naha, Napico, and the district headquarters. Since these communities were difficult to reach, researchers traveled to the communities to facilitate contact and improve the participation of the target group in the study. To locate and mobilize participants, we engaged activists who were professionals and corresponded with Community Health Agents in Brazil. Data were collected in July and August of 2017.

Upon consent, we included all patients with leprosy identified through the Murrupula Health Service reporting records and their contacts. We also included community contacts who could understand the objectives and answer questions with the help of the translator (most participants spoke the local dialect, Macua, as their main language). Only those with physical or cognitive limitations that prevented them from answering questions and undergoing physical examinations were excluded from the study.

To characterize the socioeconomic and clinical-epidemiological profiles of the patients with leprosy, semi-structured questionnaires were conducted to obtain information about the following variables: age group (under 15 years [adolescents], 15-59 years [adults] and 60 years and older [elderly]), education (primary school cycle 1 [PSC1-1^st^., 2^nd^, 3^rd^, 4^th^, and 5^th^ grade], primary school cycle 2 [PSC2-6^th^ and 7^th^ grade], secondary school [8^th^, 9^th^, 10^th^, 11^th^, and 12^th^ grade]), the number of residents in the household at the time of diagnosis, number of bedrooms in the household, and whether another resident in the household had already been diagnosed with leprosy. Data on BCG scars, the presence and number of lesions, the presence and number of affected nerves, operative classification, clinical form, degree of physical disability at the time of diagnosis, and treatment status were obtained from the clinical records.

A second form addressed the household and community contacts and included information such as sex, age, education, the number of rooms in the household, whether they were living with the index case at the time of diagnosis or not, the degree of kinship or relationship with the index case, type of living associated with the index case (whether in the same room, household, or property,) and the BCG scar. The contacts also underwent dermatological and neurological examinations to evaluate the presence of signs suggestive of leprosy, such as patches with altered sensitivity; plaques; infiltrations; physical deformities; affected nerves; operational classification; clinical form; and the degree of physical disability. These clinical details allowed the diagnosis of new leprosy cases among the interviewed contacts.

Finally, the contacts were subjected to the ML Flow and Mitsuda tests. A digital puncture was performed to collect blood for the ML Flow test, and the ML Flow rapid test from LDPTR/IPTSP/UFG - Goiás, Brazil was used. ML Flow is an immunological test that detects highly specific IgM antibodies against phenolic glycolipids (anti-PGL-I), a specific glycolipid of *M. leprae*, in both whole blood and serum samples. Five microliters of whole blood were applied to the paper base of the sample tube, and then two drops of buffer solution were added. Five minutes after the test, visual readings were taken by the investigator according to the manufacturer's guidelines. The principle of the test follows the description in the literature by Bührer-Sékula (2003)^12^.

The Mitsuda test is based on intradermal inoculation of the Mitsuda antigen, a suspension of *M. leprae* bacilli derived from heat-killed human leprosy pathogens, with a reading after 28 days, as recommended by the International Leprosy Congress in Madrid in 1953^13^. This procedure was performed as described by Doull, Guinto, and Mabalay in 1957^14^. Both tests were performed and evaluated by trained researchers.

Data were organized using the Epi Info software and Microsoft Excel. After reviewing and preparing the data, statistical analysis was performed using SPSS software, version 22.0 (IBM Corp., Armonk, NY, USA). Pearson's or Fisher's chi-squared tests were used to evaluate the relationships between the variables studied. The significance level was set at P < 0.05.

The research was approved by the Research Ethics Committee (COEP) of the Federal University of Minas Gerais (UFMG) and by the National Bioethics Committee for Health in Mozambique (REF.243/CNBS). The study conformed to ethical principles on the handling of human participants and the use of biological samples according to the Declaration of Helsinki, Brazilian Resolution 466/2013, and ethical standards applied by the National Bioethics Committee for Health (CNBS) in Mozambique.

This research was funded by CAPES through the International Program to Support Research and Teaching through International Faculty and Student Mobility-Pro-International Mobility (CAPES/AULP) - CAPES Announcement No. 33/2012. The research team consisted of two Brazilian researchers (a nurse, and a nursing student responsible for training the Mozambican team); data collection and analysis personnel; a physician responsible for leprosy diagnosis; data collection, tabulations, and analysis of the Mitsuda test; and five Mozambican nursing students involved in data collection and translation of the respondents' answers into Portuguese.

## RESULTS

During data collection, 49 patients with leprosy were diagnosed between 2013 and 2017 in the Murrupula district. Of these, 104 contacts inside and outside the index cases’ households, and 27 contacts without leprosy cases were interviewed. As shown in Table 1, 31 (63.3%) of the patients with leprosy were female, 43 (87.8%) were between 15 and 59 years of age, (the average age was 32 years), and 29 patients had little (33.95%) or no (53.6%) education.

**TABLE 1:** Socioeconomic, demographic, and clinical characteristics of 49 patients with leprosy diagnosed between 2013 and 2017. Murrupula, 2017.

Variables	N	%
**Sex**		
Female	31	63.3
Male	18	36.7
**Age Group**		
Under 15 years old	3	6.1
15 to 59 years old	43	87.8
60 and more	3	6.1
**Level of education**		
PSC1	14	33.9
PSC2	3	7.1
Secondary	3	5.4
Uneducated	29	53.6
**Number of residents in the household**		
1 to 4 people	25	51.0
5 or more people	24	49.0
**Number of rooms**		
Up to 2	46	93.9
3 or more	3	6.1
**Number of skin lesions at diagnosis**		
No	1	2.0
Less than 5	13	26.5
5 or more	35	71.4
**Injured nerves**		
Yes	18	36.7
No	31	63.3
**BCG scar**		
Yes	36	73.5
No	13	26.5
**Operational Classification**		
Multibacillary	38	77.6
Paucibacillary	11	22.4
**Physical disability at diagnosis**		
Yes	7	14.3
No	35	71.4
No Information	7	14.3
**Total**	**49**	**100**

**Source:** Prepared by the authors for the purpose of this study.

Regarding the housing and living conditions, 25 patients lived with up to four people (51.0%), and 46 (93.9%) lived in households with up to two bedrooms (Table 1).

Most of the participants (73.5%) had at least one BCG vaccine scar. The most common operational classification was multibacillary (77.6%), and 14.3% (n = 7) had some degree of physical disability at diagnosis (Table 1).

Some characteristics differed between the groups regarding contact between cases and the community. Among household contacts, the majority were female (58.7%), whereas, the community contacts were mostly male (63.0%). Although the degree of kinship with the father or mother was the most frequent in both groups, it was before spouses among household contacts (24.0%) and before siblings, and other family ties among community contacts (20.8%) were considered ( p = 0.012; Table 2).

**TABLE 2:** The association between the socioeconomic factors, type of living conditions, and clinical characteristics of 104 case contacts and 27 community contacts. Murrupula, 2017.

	Case Contacts		Community contacts		
Variables	N	%	N	%	*p-value*
**Sex**					
Female	61	58.7	10	37.0	0.053
Male	43	41.3	17	63.0	
**Age Group**					
Under 15 years old	39	37.5	4	15.4	0.069
15 to 59 years old	59	56.7	21	80.8	
60 and more	6	5.8	1	3.8	
**Level of education**					
Uneducated	47	45.2	11	40.7	0.446
PSC 1	42	40.4	9	33.3	
PSC 2	10	9.6	4	14.8	
Secondary	5	4.8	3	11.1	
**Number of rooms in household**					
Up to 2	88	85.4	21	80.8	0.552
3 or more	15	14.6	5	19.2	
**Degree of kinship with index case**					
Father/Mother	38	36.5	7	29.2	0.012
Son	9	8.7	0	0.0	
Grandparents	7	6.7	3	12.5	
Brothers and sisters	17	8.7	5	20.8	
Spouse	25	24.0	2	8.3	
Another family tie	8	7.7	5	20.8	
Neighbor	0	0.0	2	8.3	
**Contact Type**					
Household	92	81.4	9	56.3	0.030
Peridomiciliar	21	18.6	7	43.8	
BCG scar*					
Yes	75	74,3	10	40.0	0.002
No	26	25,7	15	60.0	
**Stains with altered sensitivity**					
Yes	5	4.8	10	37.0	<0.001
No	99	95.2	17	63.0	
**Confirmed diagnosis**					
Yes	4	3.8	13	48.1	<0.001
No	100	96.2	14	51.9	
Result of MLFLOW*					
Positive	9	8.8	9	33.3	0.003
Negative	93	91.2	18	66.7	
**Mitsuda test result**					
Positive	95	91.3	27	100.0	0.134
Negative	9	8.7	0	0.0	
**Total**	**104**	**100.0**	**27**	**100.0**	

**Source:** Prepared by the authors for the purpose of this study; data without information were excluded.

As shown in Table 2, the most common type of contact was household contact. However, community contacts had a higher percentage of individuals living together in the peridomicile (43.8%; p = 0.030). Sixty percent of community contacts had no inoculation scars (p = 0.002) and 37.0% (p < 0.001) had spots with altered sensitivity. The percentage of contacts diagnosed with leprosy was higher in the community contact group (48.1%; p < 0.001). The ML Flow results were also more positive (33.3%; p = 0.003).

Table 3 specifically refers to the number of contacts diagnosed with leprosy. They were predominantly men (64.7%), aged 15-59 years (76.5%), and had no schooling (52.9%). Most of the households had up to two bedrooms (76.5%).

The most common kinships were that of fathers, mothers, and siblings ( 29.4% for all categories). The type of contact within a household was the most frequently reported (52.9%), although 41.2 of respondents reported living in the house. Regarding living with the index case, 41.2% reported sleeping in the same household, which is the same percentage as those who reported living in the same household (Table 3).

**TABLE 3:** Socioeconomic factors and clinical characteristics of the 17 contacts who were living with the index case and diagnosed with leprosy. Murrupula, 2017.

Variables	Leprosy contacts	
	N	%
Sex		
Female	6	35.3
Male	11	64.7
**Age Group**		
Under 15 years old	2	11.8
15 to 59 years old	13	76.5
60 and more	2	11.8
**Level of education**		
Uneducated	9	52.9
PSC 1 (1st,2nd,3rd,4th,5th grade)	3	17.6
PSC 2 (6th,7th grade)	3	17.6
Secondary (8th, 9th, 10th, 11th, 12th grade)	2	11.8
**Number of rooms in household**		
Up to 2	13	76.5
3 or more	3	17.6
No information	1	5.9
**Degree of kinship with index case**		
Father/Mother	5	29.4
Son	0	0.0
Grandparents	1	5.9
Brothers and sisters	5	29.4
Spouse	0	0.0
Another family tie	2	11.8
Neighbor	2	11.8
No information	2	11.8
**Contact Type**		
Household	9	52.9
Peridomiciliary	7	41.2
No information	1	5.9
**Living with index case**		
Sleeps in the same room	2	11.8
Sleeps in the same household	7	41.2
Sleeps on the same ground	7	41.2
No information	1	5.9
**Scar from BCG**		
Yes	9	52.9
No	7	41.2
No information	1	5.9
**Stains with altered sensitivity**		
Yes	12	70.6
No	5	29.4
**Operational Classification**		
Paucibacillary	3	17.6
Multibacillary	14	82.4
**Clinical form**		
Tuberculoid	3	17.6
Dimorph	10	58.8
Lepromatous	4	23.5
**Degree of physical disability**		
0	2	11.8
1	9	52.9
2	6	35.3
**ML FLOW result**		
Positive	9	52.9
Negative	8	47.1
**Mitsuda test result**		
Positive	17	100.0
Negative	0	0.0
**Total**	**17**	**100.0**

**Source:** Prepared by the authors for the purpose of this study.

Nine (52.9%) contacts who were diagnosed with leprosy had at least one BCG scar and 12 (70.6%) had patches of altered sensitivity. Most were classified as multibacillary (82.4%), of the dimorphic clinical form (58.8%), while 52.9% (n = 9) experienced grade 1 physical disability, and 35.3% (n = 6) had grade 2 physical disability. Serological test results were positive in 52.9% of contacts with a positive diagnosis, and Mitsuda was positive in all cases.

## DISCUSSION

The results showed that leprosy was recognized late in all populations studied. Among the household and community contacts of patients with leprosy, 17 were diagnosed with leprosy. Of all the leprosy cases, multibacillary clinical forms with some degree of disability, skin lesions with loss of sensation, and affected nerves predominated. These findings are consistent with the results of another study in the Murrupula district, which cited difficult communication between health professionals and the population due to language issues (most participants spoke the local Macua dialect), turnover of professionals, the long duration between the onset of symptoms and diagnosis, and cultural beliefs of the population as reasons for this situation^15^.

Of note, many contacts who were diagnosed with leprosy at the time of the survey spontaneously presented for testing, with permanent physical disabilities, infiltrations, numerous skin lesions, nerve involvement, and even mutilations of the hands and feet, typical of the lepromatous pole. Considering the long incubation period of the bacillus and its clinical presentation, it is highly probable that the *M. leprae* infection occurred many years ago. This highlights an operational failure of the leprosy surveillance measures and a hidden prevalence of the disease^16^.

This epidemiological profile is not unique to the population of Mozambique. All countries with patients with leprosy, regardless of their level of endemicity, should ensure significant improvement in leprosy control, relief, and surveillance. Leprosy detection is heterogeneous and depends on the experience of the health team, as well as the population's access to health services.

For example, in 2017, the year the data were collected, and in 2018, there was an increase in leprosy diagnoses in Murrupula, with 45 and 93 cases, respectively. The cases diagnosed in 2017 experienced a lower proportion of grade 2 physical disabilities (13.3%) than the district's historical series^17^. This may reflect a possibility of improved detection of cases in the region.

A positive ML Flow test was more common among contacts diagnosed with leprosy and among the contacts of patients who presented spontaneously. Despite the limitations of serologic testing and the specifics involving the immune system, there is consensus on the association between positive anti-PGL-I serology and a higher incidence of leprosy^18^ ^,^ ^16^. There is evidence that seropositive contacts are three times more likely to contract leprosy than seronegative contacts. Therefore, serological tests have been used as markers for the degree of endemicity in a given population^19^.

In Brazil, serological tests have recently been approved for use in healthcare settings to help in the early diagnosis among contacts of patients with equivocal signs and symptoms. This is considered an advance in leprosy control, as it can facilitate the detection of individuals who are at a higher risk of contracting the disease^20^.

Regarding the Mitsuda test, the results of this study are intriguing because the classic association of negative Mitsuda test results with lepromatous leprosy was not observed^21^. All the patients of this study were positive for the Mitsuda test, including four lepromatous and 10 dimorphic cases. Classically, a positive response to the test is observed in tuberculoid patients, which reflects their ability to develop an efficient granulomatous response to *M. leprae*. In contrast, in lepromatous patients, the absence of a response to the test indicates their inability to contain bacilli multiplications, and dimorphic patients vary in their response to this test^22^.

Two possibilities to consider are nonspecific reactions to tissue residues present in the whole Mitsuda antigen produced from human leprosy, and the occurrence of secondary infections. In this case, histopathologic analysis of the induration area could clarify the presence or absence of an epithelioid granuloma with rare or absent bacilli, which is the morphological substrate of a true positive reaction^23^.

Most patients and leprosy contacts had at least one BCG vaccine scar. Currently, the BCG vaccine remains the main strategy for prophylaxis of leprosy, and the expansion of vaccination in endemic countries is strongly recommended. There was evidence of protection against *M.leprae* from 41.0% (in experimental studies) to 60.0% (in observational studies)^11^.

The expansion of vaccination in Mozambique is due to the work of the Expanded Vaccination Program, which has reduced morbidity and mortality in the child population since 1979. The BGC program achieved an 80% coverage between 1997 and 2015^24^. However, the frequency of nonvaccination was significantly higher among contacts diagnosed with leprosy and those who presented spontaneously. This finding requires attention because these populations represent spontaneous demand and late diagnosis given the clinical features presented.

Our findings suggest the importance of putting in place an active search for leprosy cases, which is often neglected in municipalities due to a lack of action planning. The search should not only take place among contacts but also in collective actions involving the entire population to make earlier diagnoses and avoid physical disabilities^16^.

In terms of socioeconomic and demographic characteristics, the majority of cases and contacts of patients with leprosy were female, which can be justified by the fact that women have a better body image, observe themselves more, and seek health services more often than men^25^. Another peculiarity of this population was that women made up 52.0% of the population in Mozambique and have a higher life expectancy^26^.

In contrast, most of the new cases diagnosed during contact examination were men, which is consistent with the literature indicating higher leprosy transmission in men^27^ ^,^ ^28^ ^,^ ^29^, late diagnosis, and a higher probability of developing physical disabilities^25^ ^,^ ^30^.

The adult age group appears to be a common category for all respondents, especially for new cases diagnosed in contacts, and is of great epidemiological importance because of the possibility that those affected may not be able to work because of physical limitations from leprosy^31^.

The high prevalence of a low educational level and a lack of schooling reflects socioeconomic vulnerability and the scenario of poor living conditions that favor the transmission of leprosy^32^ ^,^ ^33^. On the African continent, this has become even more important, given the history of deep social inequalities and exploitation of the population.

Housing conditions also illustrate the socioeconomic status of the population affected by leprosy. The majority live in small households with no more than two rooms, which causes greater proximity between the inhabitants and favors the spread of bacillus^34^ ^,^ ^16^.

Household contacts of patients with leprosy have the highest risk of contracting the disease, particularly when associated with multibacillary index cases, a lack of BCG vaccination, positive ML Flow test results, and unfavorable socioeconomic conditions^35^ ^.^ The most frequently mentioned kinship among the contact groups, including those diagnosed with leprosy, were fathers, mothers, spouses, and siblings. However, in this study, more distant relatives and neighbors were also affected. A peculiarity noted in this population was the way the houses were organized, namely, very close to each other and on the same property, which would justify the high number of people living in the proximity of the index cases.

Therefore, the search for contacts must go beyond the home and include the closest neighbors, relatives, work, and school colleagues, because it is known that social and family contacts are also at a higher risk of disease compared to the general population^36^. A study conducted in the northern region of Brazil reviewed the spatial distribution of leprosy cases diagnosed among schoolchildren, and found that all lived within 200 m of the index case^37^.

The limitations of this study include the small sample size due to the difficulties faced by the team (language difficulties and access of the communities), the complicated logistics of collecting data from the target population, and the geographic and organizational factors of the local monitoring service. The latter is also due to the sparse information on leprosy cases and their contacts.

There is strong evidence of a profile of endemic leprosy in one of the most endemic districts of Nampula Province (one of the most important districts in the country), indicating common late diagnoses and the existence of a population living in a supply vacuum. For this community, signs and symptoms suggestive of leprosy require investigations as early as possible. Additionally, the study conducted an active search among household and community contacts, so that a diagnosis could be determined at the time of the interview. The team used serological tests to contribute to the epidemiological surveillance of the disease.

It is clear, then, that much remains to be done to eliminate leprosy in Mozambique and other parts of the world. The results of this study show a tendency for late diagnoses, physical disabilities, sparse active search, and technical and operational difficulties of accessing health services. A public health policy to improve the diagnosis and care of individuals with leprosy in the region of Murrupula is warranted, and in the future, the policy should be extended to the whole country.

Our results suggest that a training program for health professionals and awareness campaigns for the general population are necessary. A publicly accessible unified database, active surveillance of leprosy cases and contacts and follow up of cases among the clinically healthy population are also important. Each leprosy case must be treated and followed up appropriately. Reference centers for the referral of specific cases and rehabilitation should also be established.

In future research, new studies that utilize serological and histopathological techniques for the epidemiological surveillance of leprosy and early diagnosis are warranted. Furthermore, the complicated logistics of access and the specificities of the region must be considered.

## References

[1] WHO - World Health Organization (2021). Global leprosy (Hansen disease) update, 2020: impact of COVID-19 on global leprosy control. Weekly epidemiological Records.

[2] Hamill LC, Haslam D, Abrahamsson S, Hill B, Dixon R, Burgess H (2019). People are neglected, not diseases: The relationship between disability and neglected tropical diseases. Trans R Soc Trop Med Hyg.

[3] Jopling WH (1991). Leprosy stigma. Lepr Rev.

[4] ILEP (2017). History of Leprosy in Mozambique.

[5] MISAU MOZAMBIQUE - Ministry of Health (2017). Leprosy.

[6] INE. National Institute of Statistics (2022). Murrupula district statistics. Maputo, 2013.

[7] Sarode G, Sarode S, Anand R, Patil S, Jafer M, Baeshen H (2020). Epidemiological aspects of leprosy. Dis Mon.

[8] Ribeiro GC, Barreto JG, Bueno IC, Vasconcelos BF, Lana FCF (2019). Prevalence and spatial distribution of Mycobacterium leprae infection in a médium endemicity municipality. Rev Rene.

[9] Silva AR, Queiroz MFA, Ishikawa EAY, Silvestre MPSA, Xavier MB (2017). Evaluation of agreement between tests for the diagnosis of leprosy. J Bras Patol Med Lab.

[10] Leturiondo AL, Noronha AB, do Nascimento MOO, Ferreira CO, Rodrigues FDC, Moraes MO (2019). Performance of serological tests PGL1 and NDO-LID in the diagnosis of leprosy in a reference Center in Brazil. BMC Infect Dis.

[11] Lastória JC, Abreu MA (2016). Brazilian Society of Dermatology against leprosy. An Bras Dermatol.

[12] Bührer-Sékula S, Smits HL, Gussenhoven GC, van Leeuwen J, Amador S, Fujiwara T (2003). Simple and fast lateral flow test for classification of leprosy patients and identification of contacts with high risk of developing leprosy. J Clin Microbiol.

[13] (1953). Memória del 6^th^ Congresso Internacional de Leprologia.

[14] Doull JA, Guinto RS, Mabalay MC (1957). Effect of BCG vaccination, lepromin testing and natural causes in inducing reactivity to lepromin and to tuberculin. Int J Lepr.

[15] Marega A, Pires PDN, Mucufo J, Muloliwa A (2019). Hansen's disease deformities in a high risk area in Mozambique: A case study. Rev Soc Bras Med Trop.

[16] Ribeiro GC, Barreto JG, Bueno IC, Costa BO, Lana FCF (2021). Uso combinado de marcadores sorológicos e análise espacial na vigilância epidemiológica da hanseníase [Combined use of serologic markers and spatial analysis for epidemiological surveillance of leprosyUso conjunto de los marcadores serológicos y del análisis espacial en la vigilancia epidemiológica de la lepra]. Rev Panam Salud Publica.

[17] Marega A, Hambridge T, Stakteas YP, Schoenmakers A, Wijk RV, Mierasd L (2022). Leprosy indicators and diagnosis delay in the Mogovolas, Meconta and Murrupula districts of Nampula Province, Mozambique: A baseline survey. Lepr Rev.

[18] Penna ML, Penna GO, Iglesias PC, Natal S, Rodrigues LC (2016). Anti-PGL-1 positivity as a risk marker for the development of leprosy among contacts of leprosy cases: Systematic Review and Meta-analysis. PLoS Negl Trop Dis.

[19] F Bernardes , Paula NA, Leite MN, Abi-Rached TLC, Vernal S, Silva MBD (2017). Evidence of hidden leprosy in a supposedly low endemic area of Brazil. Mem Inst Oswaldo Cruz.

[20] Brasil. Ministry of Health. Secretariat of Science, Technology, Innovation and Strategic Inputs in Health (2022). Systematic Review: Accuracy of complementary laboratory tests for early diagnosis of leprosy.

[21] Ridley DS, Jopling WH (1966). Classification of leprosy according to immunity. A five-group system. Int J Lepr Other Mycobact Dis.

[22] Narayan NP, Ramu G, Desikan KV, Vallishayee RS (2001). Correlation of clinical, histological and immunological features across the leprosy spectrum. Indian J Lepr.

[23] Beiguelman B (1999). Mitsuda's reaction after eighty years. Hansen. Int.

[24] Cassocera M, Chissaque A, Martins MRO, Deus N (2020). 40 years of immunization in Mozambique: A narrative review of literature, accomplishments, and perspectives. Cad Saude Publica.

[25] Ribeiro GC, Lana FCF (2015). Incapacidades físicas em casos de hanseníase: caracterização, fatores associados e evolução. Cogitare Enferm.

[26] INE. National Institute of Statistics Maputo, 2017.

[27] Barbosa CC, Bonfim CV, Brito CMG, Souza WV, Melo MFO, Medeiros ZM (2020). Spatial analysis of epidemiological and quality indicators of health services for leprosy in hyperendemic areas in Northeastern Brazil. Rev. Inst. Med. Trop. S. Paulo.

[28] Wangara F, Kipruto H, Ngesa O, Kayima J, Masini E, Sitienei J (2019). The spatial epidemiology of leprosy in Kenya: A retrospective study. PLoS Negl Trop Dis.

[29] Hierrezuelo RN, Fernández GP, Portuondo DZ (2021). Caracterización clinicoepidemiológica de pacientes con lepra en un área de salud de Santiago de Cuba. MEDISAN.

[30] Sanchez MN, Nery JS, Pescarini JM, Mendes AA, Ichihara MY, Teixeira CSS (2021). Physical disabilities caused by leprosy in 100 million cohort in Brazil. BMC Infect Dis.

[31] Lopes FC, Ramos ACV, Pascoal LM, Santos FS, Rolim ILTP, Serra MAAO (2021). Leprosy in the context of the Family Health Strategy in an endemic scenario in Maranhão: Prevalence and associated factors. Cien Saude Colet.

[32] Niitsuma ENA, Bueno IC, Arantes EO, Carvalho APM, Xavier GF, Fernandes GDR, Lana FCF (2021). Factors associated with the development of leprosy in contacts: A systematic review and meta-analysis. Rev Bras Epidemiol.

[33] Lages DS, Kerr BM, Bueno IC, Niitsuma ENA, Lana FCF (2019). A baixa escolaridade está associada ao aumento de incapacidades físicas no diagnóstico de hanseníase no Vale do Jequitinhonha. Hu rev.

[34] Cunha MHCM, Silvestre MPSA, Silva AR, Rosário DDS, Xavier MB (2017). Fatores de risco em contatos intradomiciliares de pacientes com hanseníase utilizando variáveis clínicas, sociodemográficas e laboratoriais. Rev Pan-Amaz Saude.

[35] Santos KCB, Corrêa RGCF, Rolim ILTP, Pascoal LM, Ferreira AGN (2022). Strategies for control and surveillance of leprosy contacts: integrative review. Saúde debate.

[36] Richardus JH, Oskam L (2015). Protecting people against leprosy: chemoprophylaxis and immunoprophylaxis. Clin Dermatol.

[37] Barreto JG, Bisanzio D, Frade MA, Moraes TM, Gobbo AR, de Souza Guimarães L (2015). Spatial epidemiology and serologic cohorts increase the early detection of leprosy. BMC Infect Dis.

